# The novel chemokine receptor CXCR7 regulates trans-endothelial migration of cancer cells

**DOI:** 10.1186/1476-4598-10-73

**Published:** 2011-06-14

**Authors:** Brian A Zabel, Susanna Lewén, Robert D Berahovich, Juan C Jaén, Thomas J Schall

**Affiliations:** 1 ChemoCentryx, Inc., 850 Maude Avenue, Mountain View, CA 94043, USA

## Abstract

**Background:**

Migration of metastatic tumor cells from the bloodstream into lymph nodes is thought to be facilitated by expression of the chemokine receptors CCR7, CXCR4 and, for B cell-derived tumors, CXCR5. Expression of their respective chemokine ligands (CCL19, CCL21, CXCL12 and CXCL13) by endothelial cells inside the lymph nodes facilitates the trans-endothelial migration (TEM) of these cells through high endothelial venules into the lymph node parenchyma. It is known that CXCR7, a second CXCL12 receptor, regulates TEM of CXCR4+CXCR7+ tumor cells towards a CXCL12 source. In this study, we set out to assess the potential stimulation by CXCL12 of tumor cell TEM towards other chemokines and whether CXCR7 might be able to regulate such effects.

**Methods:**

The human Burkitt's lymphoma cell line NC-37, which expresses CXCR4, CXCR5, CXCR7 and CCR7, was selected as a model system. TEM of these cells through a human HUVEC endothelial cell monolayer was used as the main model system for these studies. Regulation of their TEM behavior by various concentrations of the various cognate chemokines for the above-mentioned receptors, placed in either the source or target wells of modified Boyden chamber migration plates, was assessed by quantifying the number of cells migrated under each experimental condition.

**Results:**

Exposure of CXCR4^+^CXCR7^+ ^cancer cells to CXCL12 greatly potentiated their TEM towards the chemokines CCL19 and CXCL13. This CXCL12-potentiated TEM was inhibited by the second CXCR7 chemokine ligand, CXCL11, as well as CXCR7-specific small molecule antagonists and antibodies. In contrast, the CXCR4 antagonist AMD3100 was less effective at inhibiting CXCL12-potentiated TEM. Thus, CXCR7 antagonists may be effective therapeutic agents for blocking CXCL12-mediated migration of CXCR4^+^CXCR7^+ ^tumor cells into lymph nodes, regardless of whether the cancer cells follow a CXCL12 gradient or whether serum CXCL12 stimulates their migration towards CCR7 and CXCR5 chemokines in the lymph nodes.

## Background

Trans-endothelial migration (TEM) is a critical step in the metastatic dissemination of malignant cells from a primary tumor to distant vital organs, which is the primary cause of morbidity and mortality in cancer patients (reviewed in [1]). During metastasis, cancer cells in the bloodstream cross the endothelial cell layer of the blood vessel to enter the parenchyma of the target organ, in a manner similar to the extravasation of leukocytes. Metastasis of tumor cells to lymph nodes, whether from blood or directly via the lymphatics, is likely mediated by the same processes used by lymphocytes when they enter lymph nodes [2]. Like primary lymphocytes, tumor cells of hematopoietic and non-hematopoietic origin can express multiple chemokine receptors. CXCR4 is the most common chemokine receptor expressed by cancer cells, and has been thoroughly implicated in metastasis [3-6]. In model systems, CXCR4 regulates cancer metastasis to lymph node, bone, liver, and lung, the four most common metastatic destinations, which also express high levels of CXCL12, the only known chemokine ligand for CXCR4 [3-6]. High levels of CXCL12 are also present in the bloodstream [7-10].

CCR7, the most studied lymph node homing chemokine receptor, is expressed by certain cancer cells, in particular hematopoietic malignancies and lymph node metastases [3], as well as naïve T and B cells, while CCL19 and CCL21, the chemokine ligands for this receptor, are expressed in the T cell areas of lymph nodes [11]. Similarly, the chemokine receptor CXCR5, which guides cells to the chemokine CXCL13 present in lymph node follicles [11], has been detected on leukemia and lymphoma cells and on naïve B cells [12-15].

A poorly understood but important area of chemokine biology is the synergistic and/or inhibitory effects produced by simultaneous activation or inhibition of multiple chemokine receptors. For example, CXCL12 has been shown to potentiate the chemotaxis of CXCR4+ cells towards CCL19, CCL21 or CXCL13. In one report, CXCL12 treatment increased T cell responsiveness to CCL19 and CCL21 *in vitro *and increased CCR7-dependent recruitment of T cells into lymph nodes *in vivo *[16]. Moreover, Okada et al. showed that CXCL12-treated T cells homed more efficiently to lymph nodes and Peyer's patches than non-treated cells [17].

CXCR7 was recently identified as a second, high-affinity receptor for CXCL12 [18]. This receptor is highly expressed by a variety of cancers, including breast [19], brain [20,21], liver [22], pancreas [23], lung [24], prostate [25], melanoma [26,27] and rhabdosarcomas [28]. Like CXCR4, CXCR7 has also been implicated in tumor metastasis [24,28,29]. We recently showed that, although it does not directly mediate cell migration, CXCR7 can regulate TEM of CXCR4^+^CXCR7^+ ^tumor cells towards CXCL12, an effect that can be blocked by CXCR7-specific antagonists and the second CXCR7 chemokine ligand, CXCL11 [30]. We now describe that CXCL12 may enhance cell homing to lymph nodes by potentiating TEM towards CCL19, CCL21 and CXCL13, and show that CXCR7 can regulate this CXCL12-mediated potentiation of TEM. In the current study, we have studied the CXCL12-mediated TEM of Burkitt's lymphoma cells toward CCL19 and CXCL13. We then evaluated the involvement of CXCR7 by assessing the ability of CXCL11 and CXCR7-specific antagonists to interfere with such TEM. These studies illustrate a potential mechanism by which tumor cells metastasize to lymph nodes and other tissues, providing the rationale for antagonizing CXCR7 *in vivo *in order to block tumor metastasis.

## Methods

### Cells and reagents

The human Burkitt's lymphoma cell line NC-37 was obtained from the American Type Culture Collection (Manassas, VA). Human umbilical vein endothelial cells (HUVEC) were obtained from Lonza, Inc. (San Jose, CA), cultured according to the manufacturer's specifications, and used at passage 3. Chemokines CCL2, CCL19, CXCL9, CXCL11, CXCL12, and CXCL13 were purchased from R&D Systems (Minneapolis, MN). Anti-CCR7 (clone 150503), -CXCR3 (49801), -CXCR4 (12G5), -CXCR5 (clone RF8B2), and mouse IgG2a isotype control mAbs were also purchased from R&D Systems. Anti-CXCR7 (clone 8F11) and mouse IgG2b isotype control mAbs were purchased from BioLegend (San Diego, CA). PE-conjugated goat anti-mouse IgG was purchased from Jackson ImmunoResearch Labs, Inc. (West Grove, PA). Anti-CXCR7 (clone 11G8) mAb, mouse IgG1 isotype control mAb, CCX771 and CCX704 [30] were generated at ChemoCentryx, Inc. AMD3100 was purchased from Sigma Aldrich (St. Louis, MO). Flow cytometry was performed on a FACScan (BD Biosciences, San Jose, CA).

### Bare filter chemotaxis assay

Chemokine was diluted in chemotaxis buffer (HBSS containing 0.1% BSA), 29 μl/well was transferred to a 96-well microchamber plate, and the plate was covered with a 5 μm 96-well filter (NeuroProbe, Gaithersburg, MD). NC-37 cells were resuspended at 1 × 10^7^cells/ml in chemotaxis buffer, mixed with chemokine, antibody or compound, and 20 μl/well was added on top of the filter. The plate was incubated for 2 h at 37°C in a humidified incubator, the filter was removed, and 5 μl/well of the DNA-intercalating reagent CyQuant (Invitrogen, Carlsbad, CA) was added to the wells. Fluorescence was measured using the SpectraFluor Plus plate reader (TECAN, San Jose, CA).

### Trans-endothelial migration assay

The TEM assay was performed using 24-well plates with microporous (5 μm) transwell membrane inserts (Corning Costar Corp., Lowell, MA). HUVEC (passage 3) were suspended at 1 × 10^6^/ml in HUVEC media (Lonza), 100 μl/well was placed in the top wells and 600 μl/well of HUVEC media was placed in the bottom wells. The plate was incubated overnight at 37°C, after which the HUVEC monolayers were washed with PBS lacking Ca^2+ ^and Mg^2+^. NC-37 cells were suspended at 5 × 10^6^cells/ml in assay medium (IMDM (Invitrogen, Carlsbad, CA) with 0.1% BSA), incubated with test reagents (AMD3100, CCX704, CCX771, CXCL9, CXCL11, 11G8, mIgG1, 8F11, mIgG2b, 12G5, or mIgG2a) at room temperature for 10 min, and then added (100 μl/well) to the upper wells containing the HUVEC monolayers. In potentiated TEM experiments, CXCL12 was also added to the NC-37 cells. Assay medium containing CCL2, CCL19, CXCL12, and/or CXCL13 was then added (600 μl/well) to the bottom wells. The plates were incubated overnight at 37°C, the top wells were removed, and the cells in the bottom wells were counted.

### RNAi

HUVEC (passage 1-2) were transfected with double-stranded RNAi oligonucleotides using Lipofectamine 2000 (Invitrogen, Carlsbad, CA) according to the manufacturer's recommendations. Stealth RNAi duplex targeting CXCR7 (RNA sequence (GGCUAUGACACGCACUGCUACAUCU) and a scramble RNAi duplex control (GC content 48%) were purchased from Invitrogen. HUVEC were collected for the TEM assays three days after transfection.

## Results and Discussion

### Chemokine receptor expression on NC-37 human B lymphoma cells includes CCR7 and CXCR5

**Figure 1:**
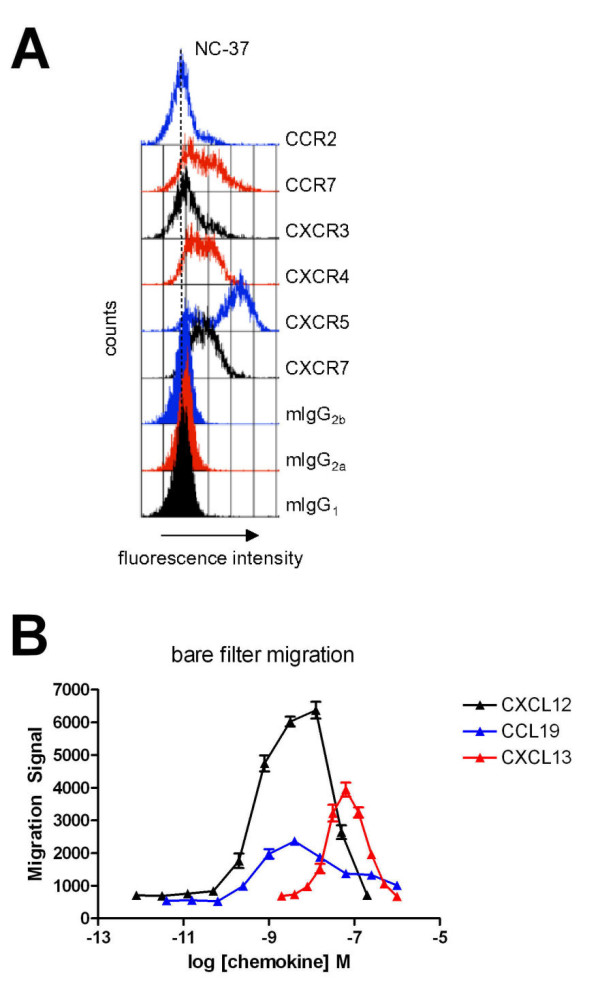
**NC-37 cells express functional CXCR5 and CCR7**. A: Flow cytometry analysis of chemokine receptor expression by NC-37 cells. Open histograms show receptor-specific staining; filled histograms show isotype controls. Relevant controls are color-matched. B: Assessment of bare filter NC-37 cell migration to a range of concentrations of CCL19, CXCL12, or CXCL13. The mean ± SEM of six wells per concentration is shown. Representative of 3 independent experiments.

To determine whether NC-37 cells are suitable to model CXCL12-mediated potentiation of CCL19 or CXCL13-driven TEM in lymph nodes, we evaluated chemokine receptor expression by these cells (Figure 1). As shown in our previous report, NC-37 cells expressed CXCR4 and CXCR7 but not CXCR3 by flow cytometry (Figure 1A and [30]). NC-37 cells also expressed chemokine receptors CCR7 and CXCR5 but not CCR2 (Figure 1A). Furthermore, NC-37 cells migrated in a dose-dependent fashion to CCL19, CXCL13 and CXCL12 in bare filter transwell chemotaxis assays (Figure 1B). Peak migration was induced by 4 nM CCL19, 60 nM CXCL13, and 10 nM CXCL12, respectively.

### CXCL12 potentiates NC-37 cell TEM to CXCL13

**Figure 2:**
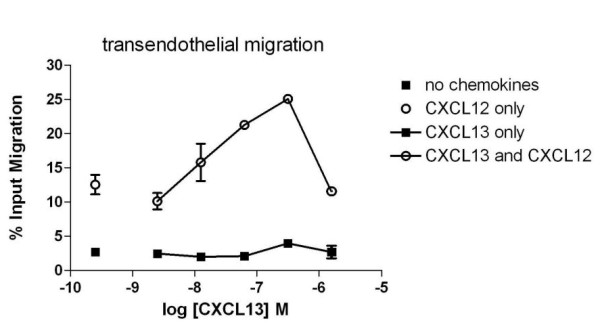
**CXCL12 potentiates NC-37 cell TEM to CXCL13**. NC-37 cell TEM to a range of concentrations of CXCL13 in the bottom well was assessed in the absence (filled black squares) or presence (open circles) of 10 nM CXCL12 in the top and bottom wells. NC-37 cell TEM in the absence of CXCL13 is indicated by a black square (absence of CXCL12) and an open circle (CXCL12 in both wells) on the left. The mean ± range of duplicate wells per condition is shown. Representative of 3 independent experiments.

In the TEM assay, NC-37 cells were tested for migration through a layer of human umbilical vein endothelial cells (HUVEC) into the bottom well of a 24-well chemotaxis plate, which contained CXCL13. Despite expressing functional CXCR5, and unlike their behavior in bare filter assays, NC-37 cells did not migrate across the HUVEC monolayer to any of the CXCL13 concentrations tested (from 3 nM to 3 μM, Figure 2). When CXCL12 was present in both top and bottom wells, however, the NC-37 cells migrated through the HUVEC monolayer in a concentration-dependent manner towards CXCL13. Peak migration was induced by 300 nM CXCL13 in the lower well, and the optimal concentration of CXCL12 to stimulate this effect was 10 nM. CXCL12 did not induce NC-37 cell TEM to the CCR2 ligand CCL2 (data not shown), indicating that CXCL12 potentiation of chemokine-driven TEM requires the presence of the specific chemokine receptor. Interestingly, the presence of CXCL12 in both wells also induced some NC-37 cell migration in the absence of other chemokines; this migration could result from increased non-directional motility (i.e. chemokinesis) of the NC-37 cells and/or increased permeability of the HUVEC monolayer.

### A CXCR7-specific compound blocks CXCL12-potentiated NC-37 cell TEM to CXCL13

**Figure 3:**
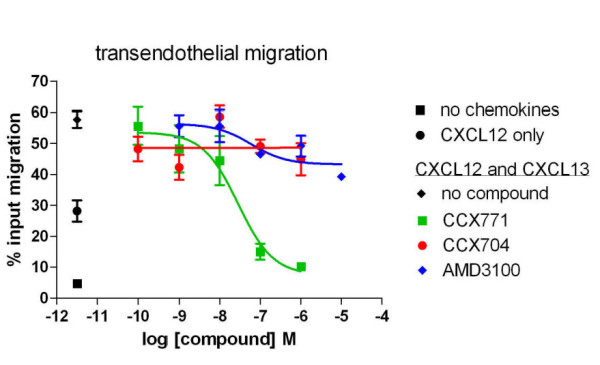
**CCX771 blocks CXCL12-potentiated NC-37 cell TEM to CXCL13**. NC-37 cell TEM to 300 nM CXCL13 in the presence of 10 nM CXCL12 in both wells was performed in the presence of a range of concentrations of CCX771 (green squares), CCX704 (red circles) or AMD3100 (blue diamonds). NC-37 cell TEM in the absence of chemokines (black square), presence of CXCL12 only (black circle) and presence of CXCL13 and CXCL12 (but absence of compounds, black diamond) are indicated on the left. The mean ± range of duplicate wells per treatment is shown. Representative of 3 independent experiments.

We previously reported that the CXCR7-specific antagonist CCX771 was 20-fold more potent than the CXCR4-specific antagonist AMD3100 in blocking NC-37 cell TEM to CXCL12 [30]. Here we evaluated the effects of CCX771 and AMD3100 in inhibiting CXCL12-potentiated NC-37 cell TEM to CXCL13 (Figure 3). CCX771 was a potent inhibitor of CXCL12-potentiated NC-37 cell TEM to CXCL13, exhibiting an IC_50 _value of 28 nM. CCX771 also inhibited the non-directional migration of NC-37 induced by CXCL12 alone, resulting in near-background levels of migration (data not shown). In comparison, AMD3100 had only a modest effect on CXCL12-potentiated TEM to CXCL13, inhibiting < 30% of cell migration. The control compound CCX704, a homolog of CCX771 with no affinity for CXCR7, had no effect on CXCL13-potentiated TEM.

### CXCR7-specific mAbs and CXCL11 block CXCL12-potentiated NC-37 cell TEM to CXCL13 or CCL19

**Figure 4:**
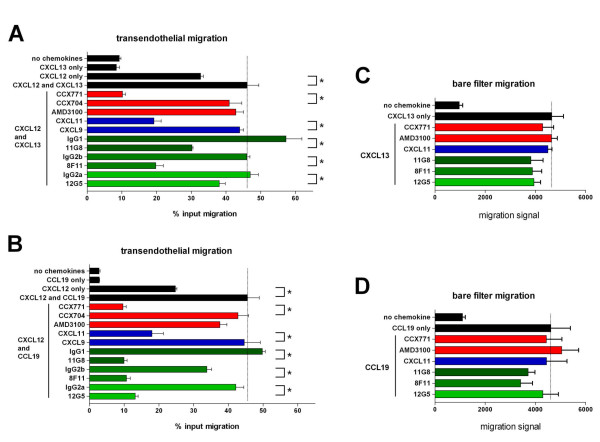
**Agents that block CXCL12 binding to CXCR7 inhibit CXCL12-potentiated NC-37 cell TEM to CXCL13 and CCL19**. A-B: NC-37 cell TEM to 300 nM CXCL13 (A) or 1 μM CCL19 (B) in the presence of 10 nM CXCL12 in both wells was performed in the additional presence of the indicated compounds (red bars), chemokines (blue bars), or mAbs (green bars). Controls (black bars) include TEM in the absence of chemokines, presence of CXCL13 or CCL19 only, presence of CXCL12 only, and presence of CXCL13 or CCL19 and CXCL12 with no other additions. C-D: NC-37 cells were analyzed in bare filter chemotaxis assays to 100 nM CXCL13 (C) or 5 nM CCL19 (D) in the presence of the indicated compounds (red bars), chemokines (blue bars), or mAbs (green bars). Controls (black bars) include chemotaxis in the absence or presence of chemokine with no other additions. * *p*-value < 0.05 by Student's *t-test *comparing the indicated treatment *vs*. matched control. For A and B, the mean ± SEM of triplicate wells per treatment is shown; for C and D, the mean ± SEM of six wells per treatment is shown. Representative of 3 independent experiments.

We next tested other agents that block CXCL12 binding to CXCR7 for their effects on CXCL12-potentiated TEM (Figure 4). The other CXCR7 chemokine ligand, CXCL11, significantly inhibited CXCL12-potentiated NC-37 cell TEM to CXCL13, while the CXCR3-specific chemokine, CXCL9, had no effect (Figure 4A). Anti-CXCR7 mAbs (clones 11G8 and 8F11) also significantly blocked potentiated migration in this assay, compared with their respective isotype controls. CCX771 was the most effective inhibitor, reducing TEM to background levels, whereas the control compound CCX704 and AMD3100 had no effect. Despite the lack of efficacy of AMD3100, the anti-CXCR4 mAb 12G5 modestly but significantly blocked CXCL12-potentiated NC-37 cell TEM to CXCL13, suggesting that CXCR4 plays a role in the TEM.

To determine whether these agents could block CXCL12-potentiated NC-37 cell TEM to other chemokines, we performed the same assay with the CCR7 ligand CCL19. Although NC-37 cells migrated to CCL19 in the bare-filter migration assay (Figure 1), the cells did not migrate to CCL19 in the TEM assay (Figure 4B). However, NC-37 cells did migrate to CCL19 in the TEM assay (optimal concentration 1 μM) when CXCL12 (10 nM) was present in both wells. As seen in the TEM assay with CXCL13, CXCL12-potentiated TEM of NC-37 cells to CCL19 was inhibited by CXCL11, CCX771, and the CXCR7 and CXCR4-specific mAbs. CXCL9, AMD3100 and CCX704 did not block migration, supporting the fact that CXCL12 potentiation of CCL19-driven TEM occurred through CXCR7.

To confirm that CCX771, CXCL11 and the mAbs did not interfere with TEM by interacting directly with CXCR5, CXCL13, CCR7, or CCL19, we tested these agents in the bare filter chemotaxis assay. CCX771, CXCL11 and the mAbs had no effect on bare filter migration of NC-37 cells to CXCL13 (Figure 4C) or CCL19 (Figure 4D), ruling out the possibility of direct inhibitory effects of these agents on CXCR5 or CCR7 or their ligands.

### CXCR7 expression by HUVEC is dispensable for CCX771 blockade of NC-37 cell TEM

**Figure 5:**
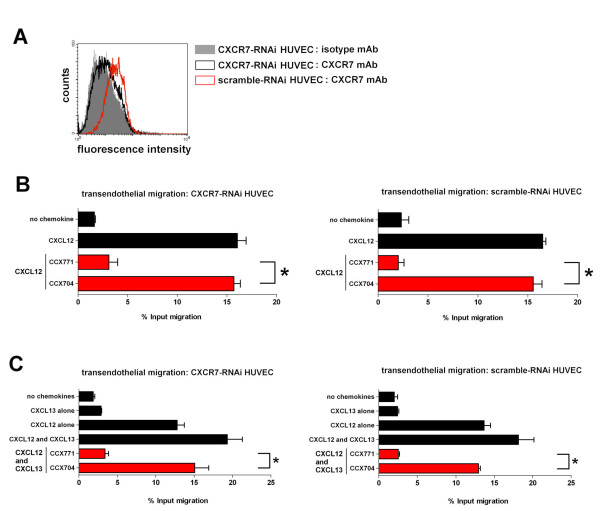
**CXCR7 expression by HUVEC is dispensable for CCX771 blockade of cell TEM**. A: HUVEC were transfected with CXCR7-targeted RNAi (black open histogram) or scramble-RNAi (red open histogram) oligonucleotides and analyzed by flow cytometry with the CXCR7-specific mAb 11G8. The filled histogram shows isotype control mAb staining. B: NC-37 cell TEM across CXCR7-targeted RNAi (left graph) or scramble-RNAi (right graph) HUVEC to 10 nM CXCL12 was performed in the presence of CCX771 or CCX704 (red bars). Controls (black bars) include TEM in the absence of CXCL12 or the presence of CXCL12 but the absence of compounds. C: NC-37 cell TEM across CXCR7-targeted RNAi (left graph) or scramble-RNAi (right graph) HUVEC to 300 nM CXCL13 in the presence of 10 nM CXCL12 in both wells was performed in the additional presence of CCX771 or CCX704 (red bars). Controls (black bars) include TEM in the absence of chemokines, presence of CXCL13 only, presence of CXCL12 only, and presence of CXCL13 and CXCL12 but the absence of compounds. * *p*-value < 0.05 by Student's *t-test *comparing the indicated treatment *vs*. matched control. For B and C, the mean ± SEM of quadruplicate wells per treatment is shown. Representative of 2 independent experiments.

We previously reported that, although the HUVEC used in the TEM assay express low levels of CXCR7, CCX771 does not inhibit TEM of CXCR4^+^CXCR7^- ^cells to CXCL12 [30]. This result, however, does not rule out the possibility that HUVEC-expressed CXCR7 plays a role in CXCL12-driven (CXCL12 in the bottom well) or CXCL12-potentiated (CXCL12 in both wells) TEM of CXCR4^+^CXCR7^+ ^cells. To address this possibility, we used RNAi methods to reduce CXCR7 protein expression in HUVEC and then evaluated the cells in the TEM assay. HUVEC transfected with CXCR7-targeted RNAi failed to stain with the anti-CXCR7 mAb 11G8 by flow cytometry, whereas HUVEC transfected with scrambled duplex RNAi retained 11G8 staining (Figure 5A). CXCR7 knockdown did not affect the ability of CCX771 to block CXCL12-driven TEM (Figure 5B) or CXCL12-potentiated TEM (Figure 5C). Therefore, the regulation of CXCL12-driven TEM and CXCL12-potentiated TEM by CXCR7 appears to occur in the migrating cells, not the endothelial cells. This result suggests that, in order to block TEM in vivo, pharmacologic antagonists of CXCR7 need only engage CXCR7 on the tumor cell, not CXCR7 on the endothelium.

In this report we demonstrate that CXCL12 can work in concert with CCL19 or CXCL13 to promote efficient TEM of CXCR7^+ ^Burkitt's lymphoma NC-37 cells. While CCL19 or CXCL13 alone did not induce TEM of these cells, which also express CCR7, CXCR4, and CXCR5, the presence of CXCL12 on the apical side or both sides of the endothelial cell layer resulted in TEM of NC-37 cells towards CCL19 or CXCL13. These observations may relate to metastasis *in vivo*, since CXCL12 is implicated in metastasis but is present both in the bloodstream and in the parenchyma of many organs (reviewed in [3-6]). Importantly, the sensitization of NC-37 cells by CXCL12 was blocked by a CXCR7-specific antagonist. Since the antagonist also blocks TEM of the NC-37 cells towards CXCL12 alone [30], we conclude that CXCR7 can regulate both CXCL12-directed TEM and CXCL12-potentiated TEM of CXCR4^+^CXCR7^+ ^lymphoma cells.

In addition to potentiating TEM towards CCL19 and CXCL13, CXCL12 alone induced TEM. That is, a subset of NC-37 cells migrated across the endothelial cell layer when CXCL12 was present at equal concentrations on both sides of the layer. This non-directed TEM was also blocked by the CXCR7 antagonist. The CXCR4-specific antagonist AMD3100 was unable to block either the non-directed TEM or the CXCL12-potentiated TEM, supporting the notion that these processes occur through CXCR7, not CXCR4. In addition, the inhibitory effects of the CXCR7 antagonist were observed regardless of whether or not the endothelial monolayer expressed CXCR7, suggesting that, *in vivo*, inhibition of TEM might require pharmacologic antagonism of CXCR7 only on the tumor cell.

Agents that target CXCR4, a chemokine receptor expressed by at least 23 different types of cancer [31], can block tumor cell TEM in mouse models *in vivo*. Treatment of mice with AMD3100 transiently reduced the seeding of i.v.-injected CXCR4^+ ^syngeneic 4T1 mammary tumor cells in the lung [32]. In a xenograft model in nude mice, treatment with AMD3100 reduced the dissemination of CXCR4^+ ^human epithelial ovarian carcinoma cells onto mesothelial cells lining the peritoneal cavity [33]. In both animal models, however, the effect on tumor cell dissemination was partial. We previously showed that AMD3100 was an inefficient inhibitor of NC-37 cell TEM to CXCL12 [30] and here we show that AMD3100 is unable to block CXCL12 potentiation of NC-37 cell TEM to CCL19 or CXCL13. Although our studies have been performed only with a lymphoma cell line and should be expanded to include other tumor types, it is possible that, for tumor cells that express both CXCR4 and CXCR7, small molecules that target CXCR7 may therefore prove to be superior agents to inhibit metastasis *in vivo*. An additional limitation of our study is the use of HUVEC cells, which might not recapitulate the behavior of specialized endothelial cells such as those found in the high endothelial venules of lymph nodes.

## Conclusions

To the extent that the chemokine CXCL12, which is found in blood and in organs commonly colonized by metastatic tumors, participates in promoting the migration of tumor cells across endothelium into those organs, our data suggest that the newly discovered CXCR7 receptor for CXCL12 may be a more effective therapeutic point of intervention than the previously studied CXCR4 receptor, which also recognizes CXCL12.

## List of Abbreviations

HUVEC: human umbilical vein endothelial cell; TEM: trans-endothelial migration.

## Competing interests

All authors (BAZ, SL, RDB, JCJ and TJS) are current or previous employees of ChemoCentryx Inc. and have equity ownership in it.

## Authors' contributions

BAZ designed and analyzed the experiments; SL conducted the majority of the experiments under the supervision of BAZ; RDB also performed some of the experiments and contributed to the preparation of the manuscript; JCJ and TJS were responsible for the overall design and analysis of the experiments. All authors have reviewed and approved the final manuscript.
